# Impact of squat set configuration on mechanical performance in paired sets of upper-body exercises

**DOI:** 10.1186/s13102-024-00912-7

**Published:** 2024-05-27

**Authors:** Danica Janicijevic, Sergio Miras-Moreno, Maria Dolores Morenas-Aguilar, Sara Chacon-Ventura, Jonathon Weakley, Amador García-Ramos

**Affiliations:** 1 Faculty of Sports Science, Ningbo University, Ningbo, China; 2 Department of Radiology, Ningbo No. 2 Hospital, Ningbo, China; 3 Department of Physical Education and Sport, Faculty of Sport Sciences, University of Granada, Camino de Alfacar, 21, Granada, 18071 Spain; 4 School of Behavioural and Health Sciences, Australian Catholic University, Brisbane, QLD Australia; 5 Sports Performance, Recovery, Injury and New Technologies (SPRINT) Research Centre, Australian Catholic University, Brisbane, QLD Australia; 6 Carnegie Applied Rugby Research (CARR) Centre, Carnegie School of Sport, Leeds Beckett University, Leeds, UK; 7 Department of Sports Sciences and Physical Conditioning, Faculty of Education, Universidad Catolica de la Santísima Concepcion, Concepción, Chile

**Keywords:** Efficiency, Resistance training, Rest redistribution, Superset, Velocity-based training

## Abstract

**Background:**

Paired sets and alternative set configurations (e.g., cluster sets) are frequently employed by strength and conditioning practitioners; however, their synergistic impact remains underexplored in research. This study aimed to elucidate whether the set configuration used in a lower-body exercise affects mechanical performance during paired sets of upper-body exercises.

**Methods:**

Twenty-one resistance-trained individuals (14 men and 7 women) randomly completed three experimental sessions that involved four sets of five repetitions at 75%1RM during both the bench press and bench pull exercises. The three experimental sessions varied solely in the activity conducted during the inter-set rest periods of each upper-body exercise: (i) *Traditional squat* – six squat repetitions without intra-set rest at 65%1RM; (ii) *Rest redistribution squat* – two clusters of three repetitions of the squat exercise at 65%1RM with 30 s of intra-set rest; and (iii) *Passive rest* – no exercise.

**Results:**

The rest redistribution set configuration allowed the sets of the squat exercise to be performed at a faster velocity than the traditional set configuration (*p* = 0.037). However, none of the mechanical variables differed between the exercise protocols neither in the bench press (*p* ranged from 0.279 to 0.875) nor in the bench pull (*p* ranged from 0.166 to 0.478).

**Conclusions:**

Although rest redistribution is an effective strategy to alleviate fatigue during the sets in which it is implemented, it does not allow subjects to perform better in subsequent sets of the training session.

**Supplementary Information:**

The online version contains supplementary material available at 10.1186/s13102-024-00912-7.

## Introduction

Resistance training (RT) offers a multitude of benefits, including enhancing muscular strength, endurance, bone density, body composition, metabolic health, and physical function, ultimately contributing to improved overall health, quality of life, and athletic performance [1]. RT-induced adaptations are heavily influenced by the manipulation of training variables such as exercise type and sequence, load lifted, volume, rest periods, and lifting tempo [2]. In a typical RT session, multiple sets of various lower- and upper-body exercises are performed, with rest periods between sets ranging from 1 to 5 min depending on the specific training goals [3]. Researchers have recommended inter-set rest periods of at least 3 min to optimize adaptations in maximal strength, maximal power, and athletic performance [4–6]. Longer inter-set rest periods (3 to 5 min) facilitate physiological and neuromuscular recovery (e.g., phosphocreatine (PCr) resynthesis), ensuring sustained high-quality performance throughout the RT session, which is known to be a critical factor for inducing training adaptations. However, to enhance training efficiency and alleviate monotony, it is feasible to integrate other physical activities during inter-set rest intervals without adversely affecting mechanical performance in the main exercises prescribed in the training session [7, 8]. This is of particular importance given the time constraints athletes and non-athletic individuals face when engaging in RT programs [9].

One alternative method of enhancing training efficiency is through the application of paired sets. Paired sets involve performing two exercises in an alternating manner with minimal rest between them and can include (i) targeting opposing muscle groups (e.g., bench press + bench pull, referred to as agonist-antagonist paired sets), (ii) engaging different limbs (e.g., bench press + squat, known as alternate-peripheral paired sets), or (iii) pairing biomechanically similar movements (e.g., barbell bench press + dumbbell bench press) [10]. While paired sets likely enhance training efficiency, their implementation may potentially impair mechanical performance when compared to the traditional RT method, which involves carrying out repetitions consecutively and with passive rest intervals provided after the completion of each set [11]. For instance, Ciccone et al. [12] reported that the inclusion of two upper-body exercises (bench press and bench pull) during rest intervals in the squat exercise resulted in compromised lower-body mechanical performance, evidenced by a reduction in repetitions to failure and average power output. Similarly, Weakley et al. [10] also reported that performing the squat exercise during rest intervals of the bench press exercise resulted in lower maintenance of velocity, power, and force compared to traditional RT methods (i.e., singular sets). Conversely, García-Orea et al. [13] found that using alternate-peripheral paired sets (bench press + squat) resulted in no differences in bar mean propulsive velocity compared to the traditional RT approach. A limitation of the latter study, despite the authors effectively monitoring the velocity of the bar to match the desired relative load (%1RM) and volume (as measured by velocity loss in the set), was that they did not adhere to a crossover design regarding the set configuration. Furthermore, it is plausible that the inclusion of relatively small independent samples (9 and 10 subjects per group) prevented the detection of significant differences, despite the number of repetitions completed in the squat exercise was 24.2% higher for the traditional-set group (30.3 ± 2.8 repetitions) compared to the alternating-set group (24.4 ± 1.7 repetitions) [13].

The above findings indicate reduced mechanical performance in alternate-peripheral paired sets relative to traditional sets [10, 13]. Additionally, it is logical to infer that the level of fatigue from the first exercise in a paired set negatively impacts the mechanical performance of the following exercise — the greater the fatigue, the more pronounced the interference. Rest redistribution set configuration, which essentially consists of distributing the repetitions of traditional continuous sets in shorter but more frequent sets, have been shown to be effective in reducing mechanical, metabolic, and perceptual fatigue levels during RT [14]. As a result, it seems plausible that using a rest redistribution set configuration in one exercise of a paired set might improve mechanical performance in the other exercise, compared to using a traditional set configuration for both exercises. However, there is also evidence that mechanical and perceptual levels of fatigue do not differ immediately after completing RT sessions based on cluster, rest redistribution, and traditional set configurations [15]. Therefore, it appears that the effects of rest redistribution set configurations on mechanical performance compared to the traditional set configuration might differ within the set (greater mechanical performance) and after a RT session (similar mechanical performance) [14, 15], while no study has examined the influence of set configuration (rest redistribution set vs. traditional set) during training involving paired sets.

The primary objective of this study was to assess the impact of the set configuration employed during the squat exercise, specifically comparing traditional and rest redistribution set configurations, on the mechanical performance of paired upper-body exercises (bench press and bench pull). Additionally, the study aims to explore whether the execution of the squat exercise, irrespective of the set configuration utilized, impacts upper-body mechanical performance when compared to traditional RT, where upper-body exercises are performed consecutively with passive rest intervals. We hypothesised that the higher mechanical performance (i.e., lower fatigue) expected for the squat exercise when performed using a rest redistribution set configuration compared to a traditional set configuration would allow for greater mechanical performance during paired sets of upper-body exercises (bench press and bench pull) [14]. Based on findings from previous studies on alternate-peripheral paired sets [10, 13], we hypothesized that traditional RT (i.e., passive inter-set rest) will yield greater mechanical performance compared to the paired set protocols implemented in this study.

## Methods

### Subjects

Twenty-one individuals, 14 males and 7 females, who were in good health and actively engaged in physical activities willingly participated in this study (Table 1). None of the participants reported any pain that could hinder their ability to perform the designated exercises, which included the bench press, bench pull, and squat. Additionally, all participants confirmed at least one year of experience with these exercises which was an inclusion criterion to participate in the study, and a skilled researcher assessed their proficiency during a preliminary session, ensuring their capability to perform the exercises with maximum effort. Subjects were explicitly instructed to abstain from any vigorous physical activity for 48 h prior to each laboratory visit and to avoid the intake of stimulant beverages, such as those containing caffeine, for at least 12 h before each testing session. Prior to the commencement of the study, the participants received detailed information about the study’s purpose and protocol, and they provided informed consent by signing a consent form. The study protocol followed the principles outlined in the Declaration of Helsinki and was approved by the Institutional Review Board of the University of (blinded for peer review).

**Table 1:** Basic characteristics of study participants

	Males (*n* = 14)	Females (*n* = 7)	Overall (*n* = 21)
Age (years)	24.0 ± 4.2	26.6 ± 9.1	24.9 ± 6.2
Body mass (kg)	80.3 ± 10.8	60.0 ± 4.2	73.5 ± 13.3
Body height (cm)	179.2 ± 8.4	162.9 ± 3.6	173.8 ± 10.6
Absolute bench press 1RM (kg)	96.2 ± 22.9	46.7 ± 7.9	79.7 ± 30.5
Absolute bench pull 1RM (kg)	90.8 ± 12.2	52.7 ± 6.7	78.1 ± 21.2
Absolute squat 1RM (kg)	113.7 ± 27.2	62.9 ± 7.8	96.8 ± 33.2
Relative to body mass bench press 1RM (kg·kg^− 1^)	1.20 ± 0.29	0.78 ± 0.13	1.08 ± 0.41
Relative to body mass bench pull 1RM (kg·kg^− 1^)	1.13 ± 0.15	0.88 ± 0.11	1.06 ± 0.29
Relative to body mass squat 1RM (kg·kg^− 1^)	1.42 ± 0.34	1.05 ± 0.13	1.32 ± 0.45

Mean ± standard deviation. 1RM, one-repetition maximum

### Design

**Fig. 1:**
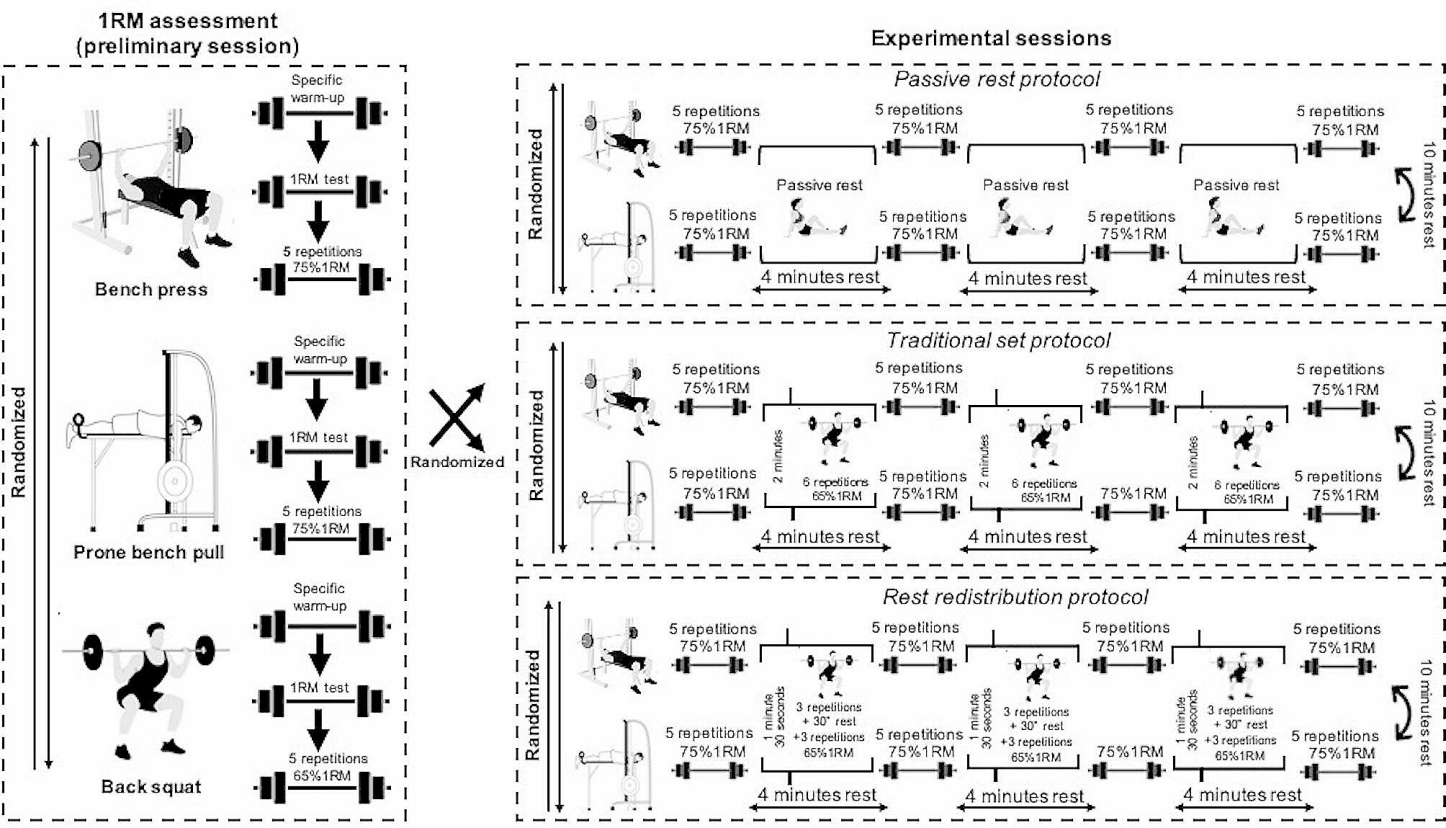
Schematic representation of the traditional squat, rest redistribution squat, and passive rest protocols

A crossover study design was used to elucidate whether the set configuration (traditional or rest redistribution) applied during sets of the free-weight back squat exercise influences the mechanical performance of paired sets that included the Smith machine bench press and Smith machine bench pull exercises (Fig. 1). Participants completed four sessions separated by 72–96 h of rest. The objective of the first session was to determine the one-repetition maximum (1RM) for the bench press, bench pull, and squat exercises. During the three subsequent experimental sessions, the subjects performed four sets of five repetitions at 75% of their 1RM for both the bench press and bench pull exercises. The three experimental sessions varied solely in the activity conducted during the 4 min that separated successive sets of both upper-body exercises: (i) *Traditional squat* – six squat repetitions without intra-set rest at 65%1RM; (ii) *Rest redistribution squat* – two clusters of three repetitions of the squat exercise at 65%1RM with intra-set rest periods of 30 s; and (iii) *Passive rest* – no physical exercise was performed. The three experimental sessions were applied in a counterbalanced order. The order of the bench press and bench pull exercises was also randomized, but the same exercise sequence was followed by individual subjects across all three sessions. Participants were instructed to perform all repetitions of the three exercises at maximal intended velocity and they received verbal velocity feedback immediately after completing each repetition to ensure maximal effort [16]. All sessions were conducted in the controlled environment of the university research laboratory, scheduled between 09:00 AM and 06:00 PM. Each participant consistently performed at the same time of day to mitigate the impact of circadian rhythms.

### Procedures

#### Preliminary session (session 1)

At the start of each session, a general warm-up routine was executed, which included jogging at a self-selected pace and dynamic stretching exercises. Subsequently, in a randomized order, subjects completed an incremental loading test during the bench press, bench pull, and squat exercises. The initial load for all exercises was set at 20 kg. The load was systematically increased by 10 kg increments until the mean velocity (MV) dropped below 0.50 m·s^− 1^ for the bench press and squat exercises or 0.80 m·s^− 1^ for the bench pull. At that point, the incremental loading test for the squat exercise was terminated, while smaller increments of 5 to 1 kg were applied for the bench press and bench pull until reaching the 1RM. The squat 1RM was estimated using the individual load-velocity relationship as the load corresponding to a MV of 0.33 m·s^− 1^ [17]. During the incremental loading test used to determine the 1RM, subjects completed two repetitions with light-moderate loads (MV ≥ 0.50 m·s^− 1^ for bench press and squat; MV ≥ 0.80 m·s^− 1^ for bench pull) and one repetition with heavier loads (MV < 0.50 m·s^− 1^ for bench press and squat; MV < 0.80 m·s^− 1^ for bench pull). Higher MV values were considered for the bench pull, as it is well-established that the MV during a 1RM trial tends to be greater for the bench pull (0.50 m·s^− 1^) than for the bench press (0.17 m·s^− 1^) and squat (0.30 m·s^− 1^) exercises [18]. The recovery time between sets was set at 3 min for light-moderate loads and 5 min for heavier loads. After completing each incremental loading test, for familiarization purposes, subjects then rested for 5 min before completing a single set of five repetitions at maximal intended velocity against either the 75%1RM for the bench press and bench pull exercises or the 65%1RM for the squat exercise. The same loads were maintained during the three remaining experimental sessions. A 10-minute interval was implemented between successive incremental loading tests. The session lasted approximately 2 h.

The bench press and bench pull exercises were performed utilizing a Smith machine (Multipower Fitness Line, Peroga, Murcia, Spain), while the squat exercise was executed with free-weights (Ruster, Jaén, Spain). During the bench press, participants maintained the 5-point contact position, ensuring contact with the head, upper back, glutes, and both feet, while employing the touch-and-go technique, which involved continuous contact between the barbell and the chest at the bottom of each repetition without pausing the weight on the chest between repetitions [19]. For the bench pull, the barbell was intentionally paused for 1–2 s on the telescopic holders of the Smith machine when both elbows reached full extension, and participants were instructed to pull the barbell until it made contact with the bottom of the bench (11.0 cm thickness). Lastly, during the back squat, participants were instructed to descend until the top of their thighs were parallel to the floor, followed by immediate execution of the lifting phase. During the squat exercise, subjects received immediate feedback after each repetition to address any instances of insufficient squat depth (visually inspected by an experienced researcher), ensuring consistent depth in subsequent repetitions across all sets. Additionally, subjects were allowed to choose their stance width in squat and grip width in bench press and bench pull according to personal preference.

#### Experimental sessions (sessions 2–4)

Prior to the commencement of each of the three experimental sessions, participants engaged in a standardised warm-up routine that encompassed jogging and dynamic stretching exercises. Following the general warm-up, a specific warm-up was conducted immediately before the working sets of the bench press, bench pull, and squat exercises. The specific warm-up for these exercises consisted of one set of 10 repetitions at 35% of 1RM, followed by three repetitions at 55% of 1RM, and finally, one repetition at 75% of 1RM. This warm-up protocol was followed by all participants except for one female, who had a bench press 1RM of 35 kg; for her, the first warm-up load represented 40% of 1RM, as the minimum load of the unloaded Smith machine barbell was 14 kg. After completing the warm-up, participants had a rest period of 3 min before commencing the first set of a upper-body exercise. A rest period of 10 min was implemented between the completion of the last set of the first upper-body exercise and the initiation of the first set of the second upper-body exercise being tested.

Throughout the three experimental sessions, participants engaged in four sets of five repetitions for each upper-body exercise at 75% of their 1RM separated by 4 min. The traditional and rest redistribution squat protocols were initiated 2 and 1.5 min after completing the sets of the upper-body exercise, respectively. To ensure comparable recovery times between the last repetition of the squat set and the first repetition of the subsequent upper-body set, different rest durations were adjusted for each protocol, taking into account the 30-second intra-set rest period specific to the rest redistribution squat protocol. During the 30-second intra-set rest periods in the rest redistribution squat protocol, the barbell was placed on the supports of the power rack. All repetitions were performed at maximal intended velocity. The MV of all repetitions was recorded by a validated linear position transducer (GymAware RS, Kinetic Performance Technologies, Canberra, Australia) which was vertically attached to the barbell of the Smith machine or to the left side of the free-weight barbell [20]. The GymAware was placed on the floor over a metallic disc to prevent it from moving during the exercise.

### Statistical analyses

The dependent variables considered in this study were (i) the mean set velocity (MSV), (ii) fastest MV of the set (MV_fastest_), (iii) MV of the last repetition of the set (MV_last_), and (iv) mean velocity decrement (MVD [%] = [MV_last_ – MV_fastest_] / MV_fastest_ × 100). Descriptive values of the dependent variables are presented as means and SD. The normal distribution of the data and the homogeneity of the variances were confirmed by the Shapiro-Wilk and Levene’s tests, respectively (*p* > 0.05). The Greenhouse-Geisser correction was used when the assumption of the homogeneity of variance was violated (*p* < 0.05). A three-way repeated-measures analysis of variance (ANOVA) with Bonferroni post hoc corrections (*set configuration* [traditional squat vs. rest redistribution squat], exercise [bench press vs. bench pull], and *set number* [set 1 vs. set 2 vs. set 3]) was applied to the MSV (average MV of the six repetitions) of the squat protocols. Another two-way repeated-measures ANOVA with Bonferroni post hoc corrections (*protocol* [traditional squat vs. rest redistribution squat vs. passive rest], and *set number* [set 1 vs. set 2 vs. set 3 vs. set 4]) was applied to MSV, MV_fastest_, MV_last_, and MVD separately for the bench press and bench pull protocols. A one-way repeated measures ANOVA with Bonferroni post hoc corrections and the Hedge’s g effect size (ES) with 95% confidence intervals were used to compare between the exercise protocols (traditional squat vs. rest redistribution squat vs. passive rest) the averaged value from sets 2–4 of MSV, MV_fastest_, MV_last_, and MVD. The scale used to interpret the magnitude of the ES was: negligible (< 0.20), small (0.20–0.49), moderate (0.50–0.79) and large (≥ 0.80) [21]. All statistical analyses were performed using SPSS software version 22.0 (SPSS Inc., Chicago, IL, USA) and statistical significance was set at an alpha level of 0.05. The figures presented in the manuscript were generated using Microsoft Excel software (Microsoft Corp., Redmond, WA, USA).

## Results

**Fig. 2:**
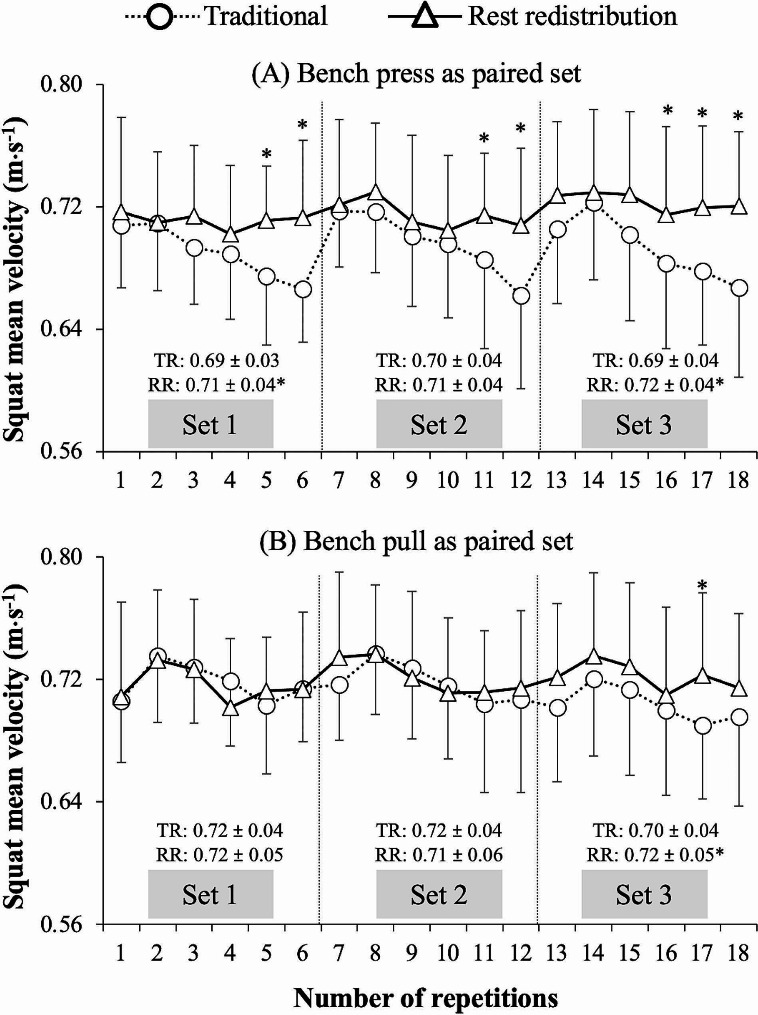
Comparison of mean velocity performance during the squat exercise performed using traditional (TR) and rest redistribution (RR) set configurations when using the bench press (Fig. 2.A) and bench pull (Fig. 2.B) as the paired exercise. The mean set velocity and the mean velocity attained at the individual repetitions are compared. *, significantly higher velocity values compared to the TR set configuration

Figure 2 shows the evolution of mean velocity through 18 repetitions (3 sets of 6 repetitions) performed in the squat exercise for each set configuration when using the bench press and bench pull as the paired exercise. The MSV of the squat exercise differed between the set configurations (F = 5.0, *p* = 0.037; greater MSV for the rest redistribution squat set configuration) and exercises (F = 7.0, *p* = 0.016; greater MSV when using the bench pull as the paired exercise), but not between the sets (F = 1.0, *p* = 0.376). The interaction set configuration × set number was significant (F = 6.4, *p* = 0.004) because the differences in MSV in favour of the rest redistribution squat set configuration were accentuated with the increase in the number of sets. The set configuration × exercise (F = 2.7, *p* = 0.116), exercise × set number (F = 2.3, *p* = 0.124), and set configuration × exercise × set number (F = 0.4, *p* = 0.645) interactions did not reach statistical significance.

**Fig. 3:**
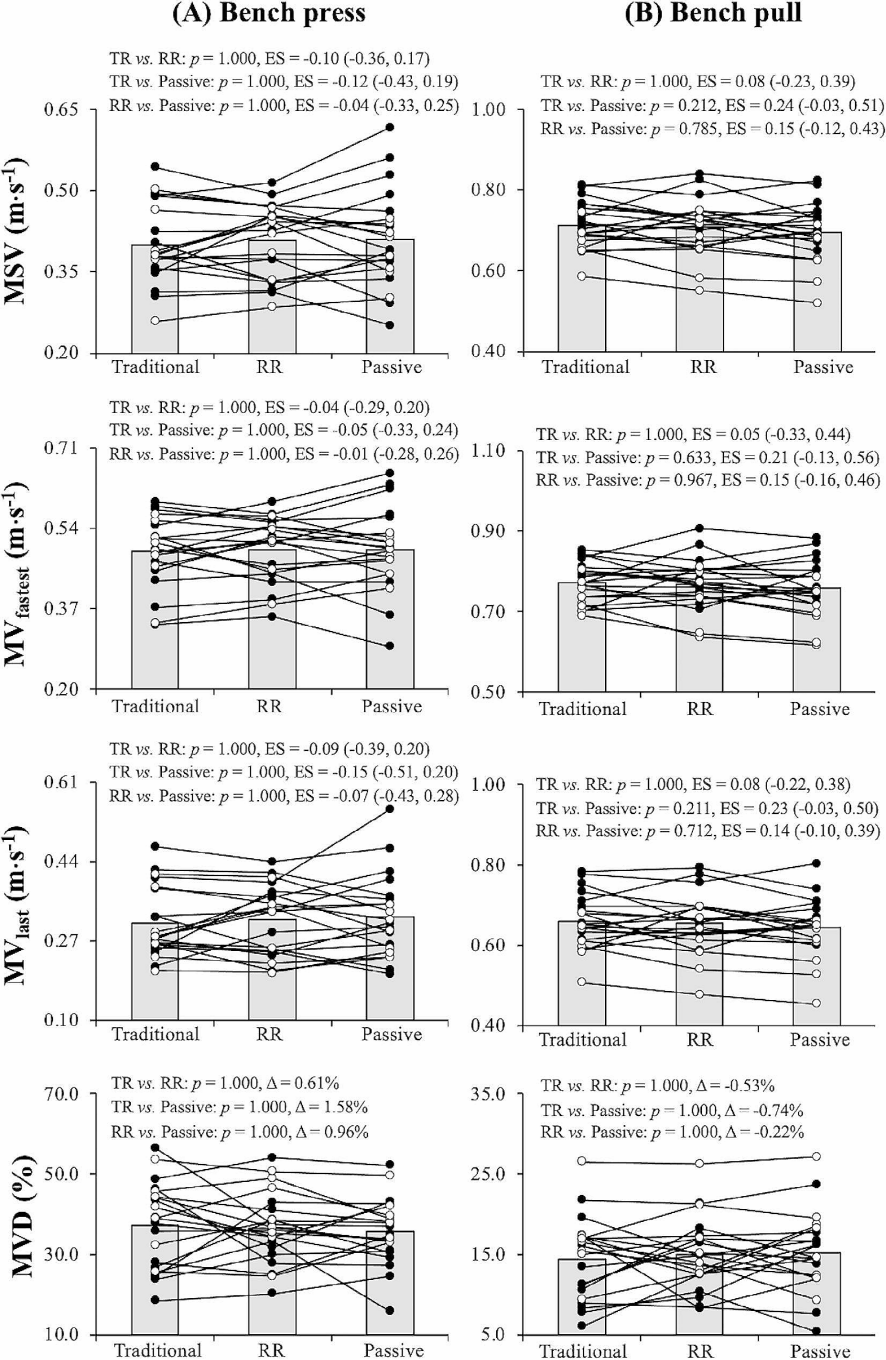
Comparison of the average value of sets 2–4 between the exercise protocols for mean set velocity (MSV; upper panels), mean velocity of the fastest repetition (MV_fastest_; middle-upper panels), mean velocity of the last repetition (MV_last_; middle-lower panels), and mean velocity decrement (MVD; lower panels) using the bench press (Fig. 3.A; upper-left panels) and bench pull (Fig. 3.B; upper-right panels) as the paired exercise. Individual values for men (black dots) and women (white dots) are depicted. ES, Hedge’s g effect size with 95% confidence intervals; TR, traditional; RR, rest redistribution. *, significant differences between the traditional squat and passive rest protocols

Table 2 presents the results of the ANOVAs conducted on MSV, MV_fastest_, MV_last_, and MVD for both the bench press and bench pull exercises. The only significant differences were detected for the main effect of set during the bench press exercise for MSV (*p* = 0.022; set 1 [0.422 m⋅s^− 1^] > set 2 [0.412 m⋅s^− 1^] > set 3 [0.402 m⋅s^− 1^] = set 4 [0.403 m⋅s^− 1^]), MV_last_ (*p* = 0.010; set 1 [0.341 m⋅s^− 1^] > set 2 [0.322 m⋅s^− 1^] > set 3 [0.314 m⋅s^− 1^] = set 4 [0.311 m⋅s^− 1^]), and MVD (*p* = 0.007; set 1 [31.8%] > set 2 [35.6%] = set 3 [36.5%] = set 4 [37.3%). Pairwise comparisons for the bench press and bench pull exercises are presented in Fig. 3.A and Fig. 3.B, respectively. The magnitude of the differences between the exercise protocols for the averaged value from sets 2–4 was always negligible for the bench press (ES < 0.20), whereas during the bench pull small differences (ES ranged from 0.21 to 0.24) in favour of the traditional squat protocol compared to the passive rest protocol were noted for MSV, MV_fastest_, and MV_last_.

**Table 2:** Two-way repeated-measures analysis of variance (ANOVA) comparing MSV, MV_fastest_, and MV_last_ of four sets of the bench press and bench pull exercises that only differed in the exercise protocol performed during the inter-set periods

Paired exercise	Variable	Protocol	Set number				ANOVA
			Set 1	Set 2	Set 3	Set 4	
Bench press	MSV (m⋅s^− 1^)	Traditional	0.42 ± 0.08	0.41 ± 0.08	0.40 ± 0.07	0.39 ± 0.08	Protocol: F = 0.5, *p* = 0.619 **Set: F = 4.5**, ***p*** **= 0.022** Protocol × Set: F = 0.7, *p* = 0.600
		RR	0.42 ± 0.08	0.41 ± 0.07	0.41 ± 0.07	0.41 ± 0.08	
		Passive	0.43 ± 0.08	0.42 ± 0.09	0.40 ± 0.09	0.41 ± 0.10	
	MV_fastest_ (m⋅s^− 1^)	Traditional	0.49 ± 0.08	0.50 ± 0.08	0.49 ± 0.08	0.48 ± 0.08	Protocol: F = 0.1, *p* = 0.875 Set: F = 0.4, *p* = 0.741 Protocol × Set: F = 0.7, *p* = 0.613
		RR	0.50 ± 0.09	0.49 ± 0.07	0.50 ± 0.07	0.50 ± 0.08	
		Passive	0.50 ± 0.08	0.50 ± 0.09	0.49 ± 0.09	0.49 ± 0.10	
	MV_last_ (m⋅s^− 1^)	Traditional	0.33 ± 0.09	0.32 ± 0.09	0.31 ± 0.07	0.30 ± 0.08	Protocol: F = 1.0, *p* = 0.361 **Set: F = 5.1**, ***p*** **= 0.010** Protocol × Set: F = 1.4, *p* = 0.222
		RR	0.34 ± 0.10	0.32 ± 0.08	0.32 ± 0.06	0.31 ± 0.09	
		Passive	0.36 ± 0.09	0.33 ± 0.10*	0.31 ± 0.08*	0.33 ± 0.10*	
	MVD (%)	Traditional	33.8 ± 11.0	36.7 ± 13.2	36.0 ± 9.1	38.9 ± 13.3	Protocol: F = 1.3, *p* = 0.279 **Set: F = 5.5**, ***p*** **= 0.007** Protocol × Set: F = 1.3, *p* = 0.264
		RR	33.4 ± 12.7	35.8 ± 11.0	35.6 ± 8.4	38.3 ± 12.3	
		Passive	28.2 ± 9.6	34.3 ± 12.0*	37.8 ± 7.9*	34.7 ± 11.0*	
Bench pull	MSV (m⋅s^− 1^)	Traditional	0.71 ± 0.06	0.72 ± 0.06	0.71 ± 0.06	0.71 ± 0.06	Protocol: F = 1.4, *p* = 0.267 Set: F = 1.3, *p* = 0.274 Protocol × Set: F = 0.4, *p* = 0.848
		RR	0.71 ± 0.07	0.71 ± 0.07	0.71 ± 0.06	0.71 ± 0.08	
		Passive	0.70 ± 0.08	0.70 ± 0.08	0.70 ± 0.07	0.69 ± 0.08	
	MV_fastest_ (m⋅s^− 1^)	Traditional	0.76 ± 0.05	0.78 ± 0.05	0.77 ± 0.04	0.77 ± 0.07	Protocol: F = 0.8, *p* = 0.478 Set: F = 1.2, *p* = 0.327 Protocol × Set: F = 1.3, *p* = 0.247
		RR	0.76 ± 0.06	0.77 ± 0.07	0.77 ± 0.06	0.76 ± 0.07	
		Passive	0.76 ± 0.07	0.75 ± 0.07	0.76 ± 0.07	0.76 ± 0.07	
	MV_last_ (m⋅s^− 1^)	Traditional	0.67 ± 0.07	0.66 ± 0.06	0.66 ± 0.08	0.66 ± 0.07	Protocol: F = 1.9, *p* = 0.166 Set: F = 1.4, *p* = 0.250 Protocol × Set: F = 0.2, *p* = 0.977
		RR	0.67 ± 0.07	0.66 ± 0.08	0.65 ± 0.07	0.65 ± 0.09	
		Passive	0.65 ± 0.09	0.65 ± 0.08	0.64 ± 0.08	0.64 ± 0.07	
	MVD (%)	Traditional	12.9 ± 5.9	15.4 ± 4.9	14.4 ± 7.1	13.5 ± 6.0	Protocol: F = 0.8, *p* = 0.475 Set: F = 2.4, *p* = 0.075 Protocol × Set: F = 1.2, *p* = 0.331
		RR	12.6 ± 4.8	15.2 ± 5.7*	15.2 ± 4.9	14.5 ± 6.2	
		Passive	14.4 ± 6.4	14.0 ± 5.6	15.9 ± 6.2	15.7 ± 5.2	

MSV, mean set velocity; MV_fastest_, mean velocity of the fastest repetition of the set; MV_last_, mean velocity of the last repetition of the set; MVD, mean velocity decline; RR, rest redistribution. *, significantly different than set 1

## Discussion

This study was designed with the main objective of elucidating whether the set configuration (traditional vs. rest redistribution) used in a lower-body exercise (squat) affects the mechanical performance during paired sets of upper-body exercises (bench press and bench pull). The degree of mechanical fatigue experienced during the sets of the squat exercise was significantly lower for the rest redistribution set configuration compared to the traditional set configuration, as evidenced by a greater MSV for the former. However, mechanical performance variables did not differ between exercise protocols (traditional squat vs. rest redistribution squat vs. passive rest) in either the bench press or the bench pull exercise. These results suggest that although rest redistribution is an effective strategy to maintain high mechanical performance during the sets in which it is implemented, it does not enhance training performance in subsequent sets of the training session.

Compared to a traditional RT approach, which involves carrying out repetitions consecutively and completing all sets for a particular exercise in succession with passive rest intervals, the weight of the scientific evidence favours the idea that paired sets cause compromised neuromuscular performance and training efficacy [10–12, 22]. However, it is important to note that the majority of the research that has led to this unfavourable perception of paired sets required participants to perform sets until they either reached or nearly reached muscular failure [10, 22, 23]. On the contrary, there is some evidence that paired sets produce similar bar execution velocity/power and volume load during non-failure RT sessions as compared to the traditional RT approach [13, 24]. In line with these findings, no significant differences were detected in the present study between the three exercise protocols (traditional squats vs. rest redistribution squats vs. passive rest) for a variety of velocity variables collected during the bench press and bench pull exercises. These findings, along with the similar adaptations in strength performance found following a 6-week non-failure RT programme using either traditional sets or paired sets with squat and bench press exercises performed at 50–70%1RM evoking 20% velocity loss [25], indicate that using paired sets is a time-efficient strategy during RT sessions based on moderate loads and submaximal levels of effort (i.e., sets terminated far from failure).

The ability of paired sets to increase RT efficiency while maintaining high mechanical outputs relies on the physical demands of the programmed exercises (e.g., load lifted, proximity to failure, or length of inter-set rest periods). As a result, it is reasonable to expect that employing set structures known to lessen fatigue during RT, such as rest redistribution or cluster set configurations, may attenuate the reduction in mechanical outputs commonly observed during paired set training schemes [14, 26]. However, contrary to our hypothesis, the set configuration used during the squat exercise did not influence mechanical performance in paired sets of the bench press and bench pull exercises. These findings appear to highlight the fact that the sudden improvement in mechanical performance that occurs when using rest redistribution set configurations—which was also seen in the current study by higher squat velocities—cannot be carried over to other exercises executed during the same RT session. This argument is also supported by the similar levels of mechanical and perceptual fatigue reported immediately following RT sessions based on the squat and bench press exercises for the cluster, rest redistribution, and traditional set configurations [15]. However, acknowledging that no significant differences were found between the paired set protocols with respect to the passive rest protocol, future studies should investigate the impact of the set configuration on mechanical performance during paired sets performed under more physically demanding training conditions.

This study described how the manipulation of set configuration (i.e., traditional or rest redistribution) of an exercise completed during the rest period of a paired set affects mechanical performance during RT. In this regard, it is crucial to emphasise that the study’s key finding—that a rest redistribution set configuration does not guarantee that subjects perform better in subsequent sets of the training session—might be falsified under different training circumstances. For instance, it is conceivable that the use of a rest redistribution set configuration might offer some advantages over the traditional set configuration when prescribing more demanding RT sessions (e.g., shorter inter-set rest periods, higher loads, or sets terminated closer to failure). Similarly, rather than involving different limbs as we did in the present study, a rest redistribution set configuration that is applied in an exercise during the recovery period may be more advantageous when pairing biomechanically similar movements (e.g., barbell bench press + dumbbell bench press) or targeting opposing muscle groups (e.g., bench press + bench pull). It is noteworthy that, when compared to the passive rest protocol, performing sets of the squat exercise, regardless of the set configuration, during the inter-set rest periods of upper-body exercises did not impair mechanical performance in the bench press or bench pull. Paired set structures, which have already demonstrated the inability to achieve mechanical performance similar to that of traditional RT methods [10, 12, 23], should be used in future research to examine how the set configuration affects mechanical performance under more fatigued conditions. While we did not conduct separate analyses for men and women due to the limited number of female participants [7], individual data for both genders were presented in Fig. 3. Upon initial examination, no significant differences between men and women were observed in mechanical performance across the three exercise protocols examined (traditional squat, rest redistribution squat, and passive rest). However, it is important to acknowledge this limitation, as potential gender-related differences in neuromuscular characteristics and fatiguability [27] as well as metabolic responses to superset training [28], may warrant further investigation.

## Conclusions

The mechanical performance during the Smith machine bench press and Smith machine bench pull exercises was unaffected by the set configuration used in the free-weight back squat exercise when it was used in the recovery period of the upper-body exercises. As anticipated, the sets of the squat exercise were performed at faster velocities using the rest redistribution set configuration than with the traditional set configuration. The reduction of fatigue during the sets of the squat exercise, however, was insufficient to predispose subjects to perform better in the following upper-body sets of the RT session. These results suggest that rest redistribution set structures cause a very brief improvement in mechanical performance, which does not affect mechanical performance in other exercises carried out during the same RT session. More research is required to clarify whether the results of the present study can be extrapolated to more physically demanding RT sessions and when other types of paired sets, such as targeting opposing muscle groups or pairing biomechanically similar movements, are used.

### Electronic supplementary material

Below is the link to the electronic supplementary material.

Supplementary Material 1

Supplementary Material 2

## Data Availability

The data and materials used to support the findings of the study are available within the manuscript or supplementary information files.
