# Supplementary material for: Impact of squat set configuration on mechanical performance in paired sets of upper-body exercises
Source: BMC Sports Sci Med Rehabil. 2024 May 27;16:119. doi: 10.1186/s13102-024-00912-7 (PMC11131196; doi:10.1186/s13102-024-00912-7)
Supplement: Supplementary file 2 — Supplementary Material 2 [file 13102_2024_912_MOESM2_ESM.docx]

**Supplementary data 1.** Individual mean velocity data obtained for all repetitions when using the bench press and bench pull as the paired exercise. Code, number assigned to the subject; Session, rest protocol implemented; Rep, repetition number.
